# A Novel End-To-End Fault Diagnosis Approach for Rolling Bearings by Integrating Wavelet Packet Transform into Convolutional Neural Network Structures

**DOI:** 10.3390/s20174965

**Published:** 2020-09-02

**Authors:** Shoucong Xiong, Hongdi Zhou, Shuai He, Leilei Zhang, Qi Xia, Jianping Xuan, Tielin Shi

**Affiliations:** 1 School of Mechanical Science and Engineering, Huazhong University of Science and Technology, Wuhan 430074, China; xiongsc@hust.edu.cn (S.X.); shuaihe@hust.edu.cn (S.H.); d201780233@hust.edu.cn (L.Z.); qxia@mail.hust.edu.cn (Q.X.); jpxuan@hust.edu.cn (J.X.); 2 School of Mechanical Engineering, Hubei University of Technology, Wuhan 430068, China; zh_hongdi@163.com

**Keywords:** fault diagnosis, bearing, deep learning, convolutional neural network, wavelet packet transform, sensor signal

## Abstract

Accidental failures of rotating machinery components such as rolling bearings may trigger the sudden breakdown of the whole manufacturing system, thus, fault diagnosis is vital in industry to avoid these massive economical costs and casualties. Since convolutional neural networks (CNN) are poor in extracting reliable features from original signal data, the time-frequency analysis method is usually called for to transform 1D signal into a 2D time-frequency coefficient matrix in which richer information could be exposed more easily. However, realistic fault diagnosis applications face a dilemma in that signal time-frequency analysis and fault classification cannot be implemented together, which means manual signal conversion work is also needed, which reduces the integrity and robustness of the fault diagnosis method. In this paper, a novel network named WPT-CNN is proposed for end-to-end intelligent fault diagnosis of rolling bearings. WPT-CNN creatively uses the standard deep neural network structure to realize the wavelet packet transform (WPT) time-frequency analysis function, which seamlessly integrates fault diagnosis domain knowledge into deep learning algorithms. The overall network architecture can be trained with gradient descent backpropagation algorithms, indicating that the time-frequency analysis module of WPT-CNN is also able to learn the dataset characteristics, adaptively representing signal information in the most suitable way. Two experimental rolling bearing fault datasets were used to validate the proposed method. Testing results showed that WPT-CNN obtained the testing accuracies of 99.73% and 99.89%, respectively, in two datasets, which exhibited a better and more reliable diagnosis performance than any other existing deep learning and machine learning methods.

## 1. Introduction

Rolling bearings are extremely vital components in rotating machinery that join the moving parts and fixing parts to maintain the normal operation of running machines. The failures of bearings can undoubtedly cause the shutdown of the whole working systems and induce other chain breaking effects, resulting in huge economical losses and occasional casualties. According to the statistics of historical rotating machinery failures, rolling bearing fault is the most commonly occurring accident that accounts for over 45 percent of failures [1]. Thus, the fault diagnosis of rolling bearing is of great importance to maximize the productivity benefits and minimize the maintenance cost.

For years, fault diagnosis in rolling bearings has been widely studied by using signal processing methods plus machine learning (ML) algorithms [2,3,4]. A typical rolling bearing fault diagnosis mainly contains three main steps: data acquisition, feature extraction and fault diagnosis. In the data acquisition step, vibration signals, motor current signals, temperature signals and acoustic emission signals are frequently used for analysis [5,6]. In the feature extraction step, statistical time domain features such as root mean square, skewness as well as kurtosis [7] and frequency domain features exposed by Fourier transform [8] are the common choices to feed to the diagnosis models. To some extent, these features can effectively distinguish the primary differences between various health conditions. However, due to the non-stationary and non-linear characteristics of the collectable signals in practical industrial applications, either time domain features or frequency domain features have their inherent limitations for completely representing the signals. Therefore, time-frequency analysis methods that can decompose the collected signals into a series of components which both contain time domain and frequency domain information are preferred in the field of fault diagnosis. Usual time-frequency analysis methods include short-time Fourier transform (STFT), wavelet transform (WT), wavelet packet transform (WPT), empirical mode decomposition (EMD) [9,10,11], etc. In the fault diagnosis step, ML algorithms are the theoretical supports for building a precise fault classification model. For example, Deng et al. [12] presented a new diagnosis approach for motor bearing which utilized empirical wavelet transform to decompose vibration signal, fuzzy entropy to compute the model input, and support vector machine to classify the fault and predict the conditions. Yan et al. [13] used an extreme learning machine to build a fault diagnosis classifier and sensitive multi-scale fault features were efficiently extracted from raw vibration signal. Li et al. [14] proposed a novel hierarchical symbol dynamic entropy to extract the fault information both in low and high frequency components and then used a binary tree support vector machine to complete the early fault diagnosis of rolling bearings. Although the ML-based fault diagnosis methods are well-developed and achieved wonderful performance, there are two deficiencies worth to consider: First, the ML-based approach inevitably needs to resort to the signal processing methods for generating discriminative features, but the manual feature extraction work needs high expertise and costs too much in terms of labor. Second, manual features extraction and selection are carefully performed based on request of the specific task, the final obtained features are not always effective when faced with the unknown working conditions or application scenarios.

Deep learning (DL), originated from the field of ML, has been found as a promising tool to automatically learn representative features from original signals [15,16]. Different from ML, DL can achieve the processes of multi-scale feature representation and final pattern recognition all at once through stacking multiple layers of information processing modules in an overall hierarchical architecture [17]. DL frameworks used in bearing fault diagnosis can be divided into three kinds: deep auto-encoder (DAE), deep belief network (DBN) and convolutional neural network (CNN). For each of them, several successful applications have been published in recent years. For instance, Chen et al. [18] used sparse auto-encoder and DBN together for multi-sensor bearing fault diagnosis, of which the former fused time and frequency domain features from different signals and the latter received fused feature vectors for further classification. Wen et al. [19] introduced a new deep transfer learning method for data-driven fault diagnosis based on sparse auto-encoder. Yu et al. [20] proposed an intelligent fault diagnosis scheme combining DBN with Dempster-Shafer theory for bearing fault conditions and fault severities classifications. Xu et al. [21] developed a novel unsupervised deep learning bearing fault diagnosis method based on DBN and a clustering algorithm. Compared with the DNN-based methods, CNN-based methods take advantage of the sparse connection, weight sharing, etc., which largely eases the training difficulty, thus, it is powerful in more complex issues. Guo et al. [22] developed a hierarchical CNN that extracted features automatically from raw vibration data and diagnosed bearing faults plus severity at the same time. Peng et al. [23] proposed a deeper residual one-dimensional CNN for adaptively learning fault features of original vibration signal meanwhile obtaining very high diagnostic accuracy for the fault diagnosis of wheelset bearings in high speed trains. Zhang et al. [24] considered the domain properties of raw vibration signals and proposed a deep CNN with wide first-layer kernels for improving domain adaptation ability and suppressing high frequency noise, promoting the accuracy of CNN-based fault diagnosis methods greatly. Khan et al. [25] developed a new dilated convolutional neural network-based deep learning model for detecting bearing faults in induction motors and achieved higher performance than conventional techniques under noisy conditions. However, noise interference and working condition variation make the original signals variegated, which increases the demand of the feature extraction ability of CNN and decreases the reliability of fault diagnosis performance. To tackle this, some domain adaptation processing algorithms are introduced. Li et al. [26] constructed a novel ST-CNN method for fault diagnosis of bearings by fusing S-transform (ST) and CNN, in which a ST layer converted sensor data into a two-dimensional time-frequency matrix and the following CNN model performed diagnosis results. Zhao et al. [27] calculated the envelope time-frequency representations of the vibration signal using Hilbert transform and synchro-squeezing transform and then built a deep CNN to learn the underlying features and determine the fault types automatically. Zhu et al. [28] presented a new bearing remaining useful life estimation method through time-frequency representation and multiscale CNN, in which the time-series signals revealed the nonstationary properties using wavelet transform. Despite converting vibration signal into time-frequency coefficient matrix having the benefits of easily exposing fault sensitive and interference-robust components, these methods also have their limitations. One is that the manual work of obtaining time-frequency information increases the overall complexity of the DL-based fault diagnosis methods, just like the manual feature extraction work does to the ML-based methods. The other is that the correlation of time-frequency information with the analyzed fault diagnosis task challenges the final accuracy of the CNN-based method.

In this paper, we proposed a novel end-to-end bearing fault diagnosis method based on WPT and CNN, named WPT-CNN. Unlike other diagnostic methods that treat the time-frequency analysis method as an independent module [26,27,28], the WPT-CCN encapsulates time-frequency decomposition and feature classification in a single network by implementing the function of WPT in the form of a modified convolutional neural layer embedded in the overall structure. WPT not only inherits the merits of WT, in that it has a good time resolution in high frequency bands and a good frequency resolution in low frequency bands, but also compensates for the shortage of WT that lacks the capacity of further decomposing the frequency components in higher frequency bands, leading to better information refinement. Many studies [29,30,31,32,33] have adopted the WPT as their preferred choice for enhancing the quality of inputs. Compared to other existing WPT-based studies, a unique point of our proposed WPT-CNN is that the WPT function in the network is self-adaptive to the request of specific tasks, since the layer that achieves the WPT function can be also trained by gradient descent backpropagation optimizing algorithms. The optimizing direction of the wavelet filter coefficients is towards higher fault classification accuracies, so it does not need to worry that the alteration of the coefficients destroys the validity of the WPT. Instead, the coefficients could appropriately learn the characters of the dataset during the optimizing procedure, resulting in a better generalization performance. Based on the above, the WPT-CNN could surpass other time-frequency analysis-based bearing fault diagnosis methods in two aspects: First, it could achieve higher and more robust diagnosis accuracies than other methods. Second, it could directly receive original signal data as input while other methods need to transform signals into time-frequency matrices ahead of time.

The remainder of this paper is summarized as follows. Section 2 overviews the theories of CNN and WPT. Section 3 describes the framework of the proposed WPT-CNN-based bearing fault diagnosis method. In the Section 4, the performance of the raised method is verified by experiments on two rolling bearing fault test rigs. Finally, Section 5 provides the discussion.

## 2. A Brief Theoretical Background

### 2.1. Convolutional Neural Network

Convolutional neural network (CNN) [34] is a variant of artificial neural network (ANN) which has the merits of sparse connection, weight sharing, etc. Compared to traditional neural networks, CNN greatly reduces the number of trainable parameters, thus enabling deeper network architecture. A CNN mainly consists of three kinds of layers: convolutional layer (Conv-Layer), pooling layer (Pool-Layer) and fully connected layer (FC-Layer).

**Figure 1:**
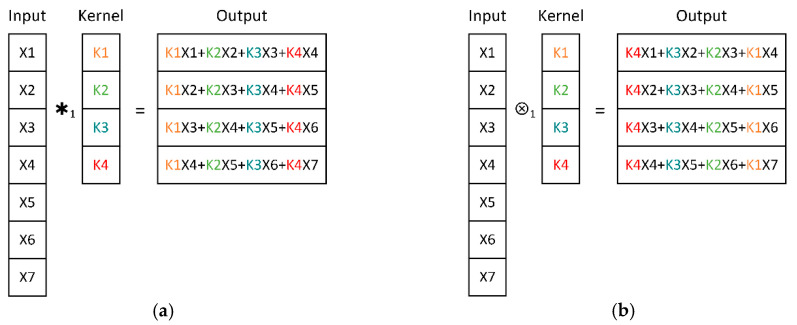
One-dimensional convolution operation in: (**a**) a convolutional neural network CNN; (**b**) mathematical definition.

Conv-Layer is the main component in a CNN which “convolves” input matrices with several trainable kernels and generates relevant feature maps. Every output neuron is activated by the partial input values due to the kernels’ limited volume, imposing the Conv-Layer concentrated on extracting robust features from the small-sized input sub-regions, which are known as the local receptive field [35]. With the layers stacking, upper Conv-Layers naturally extend the local receptive field and extract features more abstract and comprehensive, which can be finally used for decision making. It is worth noting that the so-called “convolution” operation in a Conv-Layer is actually the cross-correlation mathematical operation, but it was named as such for convenience because of the similarity with convolution operations. Figure 1 shows the one-dimensional convolution operations in CNN and the mathematical definition, respectively, from which it can be seen that one can easily convert any operation to the other by inversing the order of kernel weights. Supposing that the input matrix is $x\in R^{m\times m}$, the kernel is $k\in R^{k\times k}$, the convolution stride is s∈R, and the output matrix is $a\in R^{n\times n}$, here n=⌈(m−k+1)/s⌉ and ⌈·⌉ is the round-up function, the convolution operation in CNN can be defined as:

(1) $$\{\begin{array}{l} \;\;\;a=x{*}_{s}k\\ a_{i,j}=\sum_{p=0}^{k-1}\sum_{q=0}^{k-1}k_{p,q}x_{is+p,js+q},\forall i,j \end{array}$$

where “${*}_{s}$” denotes the convolution operation in CNN with stride s, $a_{i,j}$ is the ith row, jth column element of output a, $k_{p,q}$ is the pth row, qth column element of kernel k, $x_{is+p,js+q}$ is the (is+p)th row, (js+q)th column element of the input x. Similarly, the formal convolution operation in math definition can be expressed as:

(2) $$\{\begin{array}{l} \;\;\;a=x{*}_{s}k\\ a_{i,j}=\sum_{p=0}^{k-1}\sum_{q=0}^{k-1}k_{p,q}x_{is+p,js+q},\forall i,j \end{array}$$

where *“*${⮾}_{s}$*”* denotes the formal mathematical convolution operation with stride s. In Equation (1) and Equation (2), if the second dimensions of input and kernel are set to 1, the convolution operations of one-dimensional edition can be available.

Acting like a special kind of Conv-Layer, Pool-Layer only includes one parameter-free down-sampling kernel and traverses the input feature maps with big steps. The function of Pool-Layer is to remarkably reduce the redundant information and enlarge the receptive field, aiming for enhancing the robustness and performance of the model [36]. Denoting $x\in R^{m\times m}$ as input matrix, k with shape of k×k as down-sampling kernel, s∈R as pooling stride and $a\in R^{\frac{m}{s}}$ as output matrix, the pooling process of Pool-Layer can be expressed as:

(3) $$\{\begin{array}{l} \;\;\;a=x⇓k\\ a_{i,j}=\mathrm{DS}\left(x_{is,js},x_{is+1,js},x_{is,js+1},\ldots ,x_{is+k-1,js+k-1}\right),\forall i,j \end{array}$$

where “⇓” denotes the down-sampling operation, DS(·) denotes the down-sampling function contained in the kernel k, e.g., max function. In this paper, Max Pool-Layer and Global Average (GA) Pool-Layer [37] are adopted.

FC-Layer is a classical network layer structure that exists in the most neural networks. In CNN, FC-Layers are usually the uppermost network layers which act as a classifier [36]. Denoting the input vector as $x\in R^{m}$ and the output vector as $a\in R^{n}$, the output of a FC-Layer can be calculated as:

(4) $$\{\begin{array}{l} \;a=x\cdot w+b\\ a_{i}=\sum_{j=0}^{m-1}x_{j}w_{j,i}+b_{i},\forall i \end{array}$$

where $w\in R^{m\times n}$ is the weight matrix, $b\in R^{n}$ is the bias vector, $w_{j,i}$ is the jth row, ith column element of matrix w and $b_{i}$ is the ith element of bias vector b. Regardless of the Conv-Layer or the FC-Layer, the outputs are passed through an ReLU [38] activation function for introducing the nonlinearities.

### 2.2. Wavelet Packet Transform

In vibration-based fault diagnosis applications, the properties of signals are easy to be distorted by varying operating conditions and strong noise background, which calls for time-frequency analysis methods [39]. Wavelet transform (WT), known as mathematical microscope, is an ideal time-frequency localization tool for nonstationary signal processing. As any transform, the WT aims to project the signal to another domain in which richer information can be revealed in an easier way, and the inverse WT allows the signal to reconstruct back to the original domain. In many cases, the inverse transform is not needed and the transformed data can be treated as new features for further processing [40]. Unlike Fourier transform whose basis functions are sine and cosine, which are only local in the frequency domain, wavelets are well-localized in both time and frequency domains. With the character of wavelet basis function, WT can be very effective to describe signals with sharp spikes and discontinuities.

The realization of WT can be divided into the continuous WT and the discrete WT. Since any real-world data processed on computers must be a discrete signal, discrete WT is preferable in this paper. Given a sampled data sequence $f\in R^{m}$ (where $m=2^{N}$) and an orthogonal wavelet W, the WT of the data can be presented as [40]:

(5) $$\{\begin{array}{c} a=f{⮾}_{2}w_{l}\\ d=f{⮾}_{2}w_{h} \end{array}$$

where $a\in R^{\frac{m}{2}},d\in R^{\frac{m}{2}}$ are the approximation coefficients and the detail coefficients at the next decomposition level, respectively, ${⮾}_{2}$ denotes the formal mathematical convolution operation with stride 2. Before WT, the data sequence f is padded with some numbers at two ends to match the output dimension. $w_{l}$ and $w_{h}$ are called the low-pass filter and high-pass filter, respectively, and are specified by the wavelet W. Once a wavelet has been selected, the two filters can be defined by the same set of wavelet filter coefficients (WFCs):

(6) $$\{\begin{array}{l} \;w_{l}=\left[c_{0},c_{1},c_{2},\ldots ,c_{2k-3},c_{2k-2},c_{2k-1}\right]\\ w_{h}=\left[c_{2k-1},-c_{2k-2},c_{2k-3},\ldots ,-c_{2},c_{1},-c_{0}\right] \end{array}$$

where $c_{i}$ is the *i*th low-pass filter coefficient and 2*k* is the length of the two filters.

**Figure 2:**
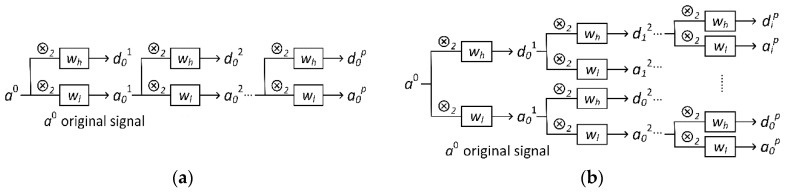
The decomposition tree of (**a**) wavelet transform; (**b**) wavelet packet transforms.

Multilevel WT can be applied through the “tree algorithm” [41] to offer a hierarchical and multiresolution representation of signals, as shown in Figure 2a. However, the tree algorithm only further decomposes the approximation coefficients at each level of resolution but leaves the detail branch unused. As an extension of the WT, wavelet packet transform (WPT) applies the transform in Equation (5) recursively to both the approximation coefficients and the detail coefficients, resulting in an expansion of tree structure of the multilevel WT, as shown in Figure 2b. Through WPT, the signal can be transformed to several uniform sets of wavelet packet coefficients, which is more suitable than WT for processing by the neural networks.

## 3. Proposed Fault Diagnosis Framework

As discussed in the Section 2.2, the time-frequency analysis method is very necessary in fault diagnosis as it has the merits of providing reliable time-frequency features as well as dividing useful and noised information of signals into different frequency bands. However, processing signal with the time-frequency analysis method also adds an extra step in the procedure of fault diagnosis, which brings some inconvenience in real applications. In this paper, which considers the effectiveness of WPT and its similar implement details with neural layers, we propose an intelligent end-to-end bearing fault diagnosis method named WPT-CNN, which can fuse time-frequency analysis ability into the architecture of the classification model and automatically search for more suitable time-frequency coefficients during the training process.

### 3.1. Implementing WPT with Conv-Layer

**Figure 3:**
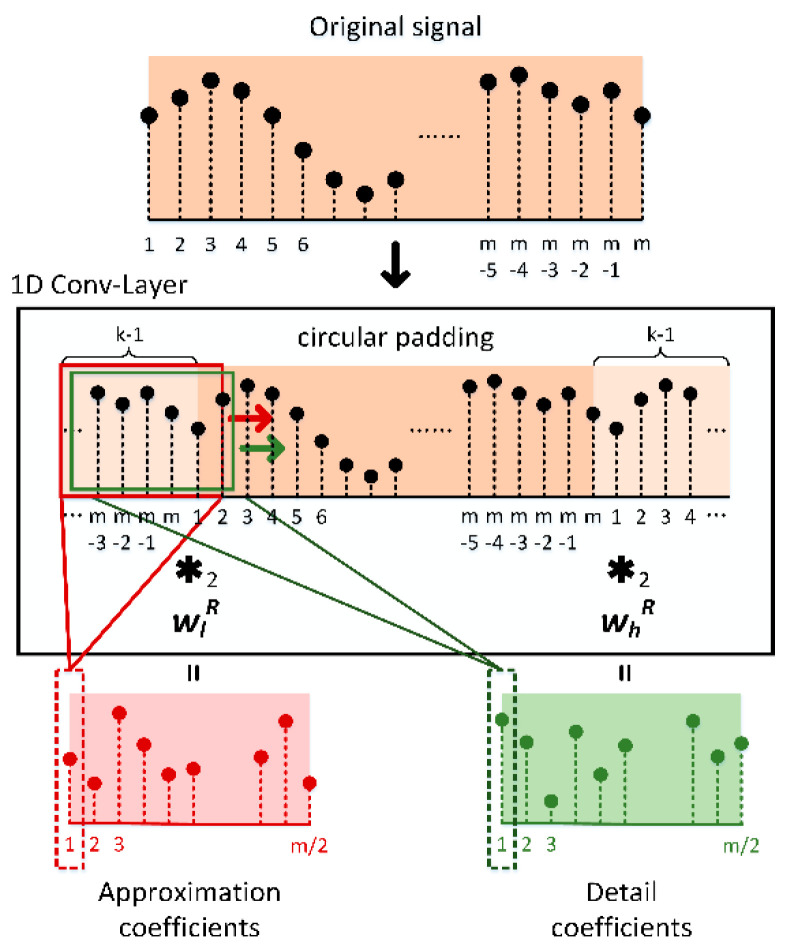
Single-level wavelet packet transform implemented in the 1D convolutional layer (Conv-Layer).

**Figure 4:**
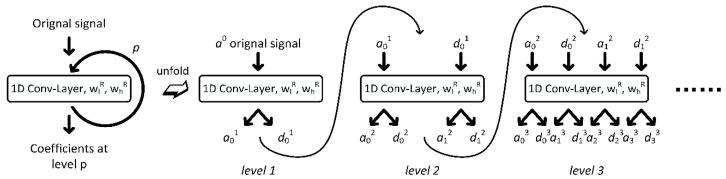
Recursive 1D Conv-Layer for multilevel wavelet packet transform.

From Equation (2) and Equation (5), it can be found that the wavelet packet coefficients are obtained by the data sequence convolved with the two wavelet filters. Additionally, Figure 1 indicates how to transform the “convolution” in a Conv-Layer to a formal convolution operation. Supposing that a wavelet basis function has been defined with the filter coefficient length of 2k, then a single-level wavelet packet transform can be expressed as the form in Figure 3. Note that the $w_{l}^{R}$ and $w_{h}^{R}$ are the inversion of the two wavelet filters in Equation (6). For achieving a multilevel wavelet packet transform as shown in Figure 2b, one can modify the 1D Conv-Layer in Figure 3 into a recursive version, which continuously feeds the original output back into the structure and keeps generating the next level of decomposition coefficients, as shown in Figure 4. The recursive 1D Conv-Layer is marked as WPT-Layer, in which only one set of kernel parameters is truly existed. For the WPT-Layer it has been proved that with a given set of WFCs, it produces the same calculation results as the “*wpdec*” function with periodical mode in the MATLAB software.

### 3.2. Overall WPT-CNN Architecture

**Figure 5:**
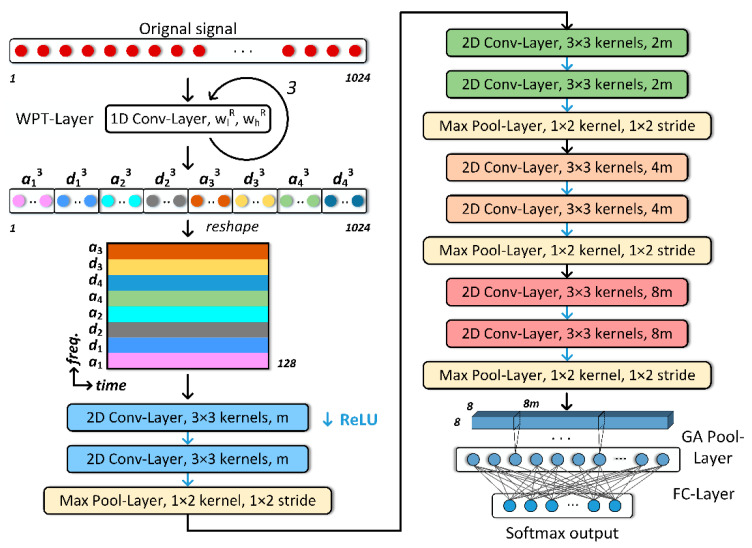
Proposed WPT-CNN architecture (example for 1024 data points of input signal and three levels of WPT).

With the help of WPT-Layer, the proposed WPT-CNN can directly receive raw vibration signals as inputs. The overall architecture of WPT-CNN is illustrated in Figure 5. Compared to exploring fluctuation patterns from a raw data sequence as features, WPT-Layer provides extra frequency information to the following layers and reduces the difficulty of extracting features by splitting complex information of signal into various frequency bands, which is beneficial for enhancing the performance of the model. After sets of coefficients have been acquired, they are reshaped to a two-dimensional (2D) time-frequency matrix for further extraction. A notable detail is that to arrange the coefficient in the order of frequency bands, every node needs to be sorted according to the index of binary Gray code. The consideration of treating frequency as an independent dimension instead of the channel of feature map is direct because the summation of the whole frequency information makes WPT meaningless. After the WPT-Layer, several 2D Conv-Layers with 3 by 3 convolution kernels are followed to extract features from brief to abstract, and between each two Conv-Layers, a Max Pool-Layer with 1 by 2 pooling kernel is inserted to reduce the number of output neurons only along the time axis. Zero-padding is utilized in 2D Conv-Layers to maintain the output feature map size equal to the input size. GA Pool-Layer plays a role in transforming 2D feature maps into 1D vectors and a subsequent FC-Layer calculates the weighted scores of every class to the softmax output layer. During the FC-Layer, dropout [42] technique is used to prevent overfitting and provide an improved result ensemble.

A huge advantage of the proposed WPT-CNN model is that the WFCs in WPT-Layer are trainable, which implies the wavelet basis function can be self-adaptive. It is known that selecting a proper wavelet function requires skill because the fit between the signal and wavelet function often influences the accuracy of fault diagnosis. For any previous application that processes vibration signals with WPT, the predefined wavelet basis function is fixed and there is no exact idea to validate the appropriateness of the selection. But the WPT-CNN overcomes this deficiency by fine-tuning the WFCs to learn the dataset properties using a gradient descent backpropagation algorithm and raw vibration signals. Since the WFCs can automatically optimize to more appropriate situations during training, the performance of WPT-CNN can be improved freely and initialized wavelet function can be selected less rigorously. Moreover, training deep neural networks is a non-convex optimizing problem and the model initial states have a lot of influence on the final converged local minimums. Sometimes touched local-minimums may not fit in the character of time-frequency matrices from a specific wavelet basis function, causing a little performance deterioration. Fine-tuning the WFCs together with the other layer parameters can remedy this discrepancy and improve the coordination of the whole network structure, ensuring a more reliable high-performance fault diagnosis.

### 3.3. Details of Training WPT-CNN

When the WFCs are fixed, WPT-Layer can be thought of as a constant WPT data processing module and the rest of WPT-CNN is the normal 2D CNN structure for category classification. Additionally, if the WFCs are unfrozen to be trainable, WPT-Layer becomes a standard 1D Conv-Layer and the WPT-CNN are still capable of training with a gradient descent backpropagation algorithm. As mentioned in Section 3.1, WPT-Layer only contains one set of trainable parameters which is shared by two convolution kernels. To make this happen, denoting the two kernels in WPT-Layer as $k_{1}$ and $k_{2}$, they can be expressed in the form

(7) $$\{\begin{array}{l} k_{1}=w_{l}^{R}=\left[c_{2k-1},c_{2k-2},c_{2k-3},\ldots ,c_{2},c_{1},c_{0}\right]\\ k_{2}=w_{h}^{R}=k_{1}\cdot T\\ T=\left[\begin{array}{ccc} \begin{array}{cc} 0 & 0\\ 0 & 0\\ 0 & 0 \end{array} & \cdots & \begin{array}{ccc} 0 & 0 & 1\\ 0 & -1 & 0\\ 1 & 0 & 0 \end{array}\\ \vdots & \ddots & \vdots \\ \begin{array}{cc} 0 & 1\\ -1 & 0 \end{array} & \cdots & \begin{array}{ccc} 0 & 0 & 0\\ 0 & 0 & 0 \end{array} \end{array}\right] \end{array}$$

where $c_{i}$ is the *i*th wavelet filter coefficient in Equation (6). Now that the kernel $k_{2}$ can be transformed by the kernel $k_{1}$ multiplying with the constant matrix T, it can also be said that WPT-Layer only includes one trainable convolution kernel.

For a single-label classification task, cross-entropy loss is the typical loss function that can accelerate the converging speed of the model weights. In this paper a special penalty term is also added to the cross-entropy to constrain the L2 norm of post-optimized WFCs roughly equal to one, which is necessary for maintaining the decomposition ability of WPT-CNN. A weight decay schedule is applied at the gradient update step as done in [43] and canceled on the WPT-Layer because the WFCs have their own regularization. The overall loss function applied to train WPT-CNN is defined as:

(8) $$L\left(p\left(x\right),q\left(x\right)\right)=-\sum_{i=1}^{C}p_{i}\left(x\right)\cdot \log\left(q_{i}\left(x\right)\right)+\alpha {\left(1-\|k_{1}\|{{}_{2}}^{2}\right)}^{2}$$

where C is the number of the classes, $p_{i}\left(x\right)$ and $q_{i}\left(x\right)$ are the real and the estimated probability of the input *x* belonging to the *i*th class, respectively, ***α*** is a hyperparameter controlling the strength of the penalty term and $k_{1}$ is the kernel in Equation (7).

During training we need to propagate the gradient of loss L(p(x), q(x)) back through the WPT-Layer, as well as compute the gradients with respect to the parameters of this layer. For simplicity, denoting $k\in R^{2k}$ as the only trainable kernel in WPT-Layer, $\bar{k}\in R^{2k}$ as its transformed form, $a^{0}\in R^{m}$ as the input signal, p∈R as the maximal decomposition level and $a_{i}^{l},d_{i}^{l}\in R^{t}\;\left(i=0,1,\ldots ,2^{l-1}-1;t=m/2^{l}\right)$ as the *i*th approximation coefficient sequence and detail coefficient sequence at the *l*th level, respectively, the gradient of the kernel *k* can be calculated as follows, using the chain rule:

(9) $$\begin{array}{l} \mathrm{Define}:\forall f=\left[f_{0},f_{1},\ldots ,f_{m-2},f_{m-1}\right]\in R^{m},\\ \;\;\;\;\;\;\;\;\;\;\;\;\;\;f^{\mathrm{fp}}=\left[f_{m-k},\ldots ,f_{m-1},f_{0},f_{1},\ldots ,f_{m-2},f_{m-1},f_{0},\ldots ,f_{k-2}\right],\\ \;\;\;\;\;\;\;\;\;\;\;\;\;\;f^{\mathrm{bp}}=\left[\overset{2k-1}{\overbrace{0,\ldots ,0}},f_{1},0,f_{2},0,\ldots ,0,f_{m-1},0,f_{m},\overset{2k-1}{\overbrace{0,\ldots ,0}}\right]\\ \mathrm{Define}:\frac{\partial L}{\partial x}=\mathrm{grad}\left(x\right)\\ \begin{array}{cc} 1.\{\begin{array}{l} \;\delta_{i}^{l-1}=\mathrm{grad}{\left(a_{2i}^{l}\right)}^{\mathrm{bp}}{*}_{1}k^{R}+\mathrm{grad}{\left(d_{2i}^{l}\right)}^{\mathrm{bp}}{*}_{1}{\bar{k}}^{R}\\ \psi_{i}^{l-1}=\mathrm{grad}{\left(a_{2i+1}^{l}\right)}^{\mathrm{bp}}{*}_{1}k^{R}+\mathrm{grad}{\left(d_{2i+1}^{l}\right)}^{\mathrm{bp}}{*}_{1}{\bar{k}}^{R} \end{array} & l=2,3,\ldots ,p \end{array}\\ 2.\{\begin{array}{l} \begin{array}{cc} \mathrm{grad}{\left(a_{i}^{l-1}\right)}_{j}=\{\begin{array}{c} \delta_{i}^{l-1}{}_{j+k}\;\;\;\;\;\;\;\;\;\;\;\;\;\;\;\;\;\\ \delta_{i}^{l-1}{}_{j+k}+\delta_{i}^{l-1}{}_{j+k-\mathrm{sgn}\left(j-k\right)\cdot t} \end{array} & \begin{array}{c} k-1\leq j<t-k+1,\\ else \end{array} \end{array}\\ \begin{array}{cc} \mathrm{grad}{\left(d_{i}^{l-1}\right)}_{j}=\{\begin{array}{c} \psi_{i}^{l-1}{}_{j+k}\;\;\;\;\;\;\;\;\;\;\;\;\;\;\;\;\;\\ \psi_{i}^{l-1}{}_{j+k}+\psi_{i}^{l-1}{}_{j+k-\mathrm{sgn}\left(j-k\right)\cdot t} \end{array} & \begin{array}{c} k-1\leq j<t-k+1,\\ else \end{array} \end{array} \end{array}\\ 3.\mathrm{grad}\left(k\right)=\sum_{l=1}^{p}\sum_{i=0}^{2^{l-1}-1}[{a_{i}^{l-1}}^{\mathrm{fp}}{*}_{1}\mathrm{grad}\left(a_{2i}^{l}\right)+{d_{i}^{l-1}}^{\mathrm{fp}}{*}_{1}\mathrm{grad}\left(a_{2i+1}^{l}\right)\\ \;\;\;\;\;\;\;\;\;\;\;\;\;\;\;\;\;+\left({a_{i}^{l-1}}^{\mathrm{fp}}{*}_{1}\mathrm{grad}\left(d_{2i}^{l}\right)+{d_{i}^{l-1}}^{\mathrm{fp}}{*}_{1}\mathrm{grad}\left(d_{2i+1}^{l}\right)\right)\cdot T^{-1}]\\ \;\;\;\;\;\;\;\;\;\;\;\;\;\;\;\;\;+4\alpha \left({\left\|k\right\|}_{2}{}^{2}-1\right)\cdot k \end{array}$$

where $k^{R}$ and ${\bar{k}}^{R}$ are the inversion of the k and $\bar{k}$, respectively, “${*}_{1}$” is the convolution operation with stride 1 in Equation (1), T^-1^ is the inverse matrix of T in Equation (7). From Equation (9), it can be seen that the gradients of loss L(p(x), q(x)) with respect to k are the summation of so many additive terms that are exponentially related with the number of maximal decomposition level p. Since the calculation of gradient with respect to k is part of the network training procedure when the WPT-Layer is unfrozen, the choice of number of decomposition level p is worth considering to speed up the training. To trade off the training speed and the decomposition precision, p is selected to be equal to 3 in this paper.

**Figure 6:**
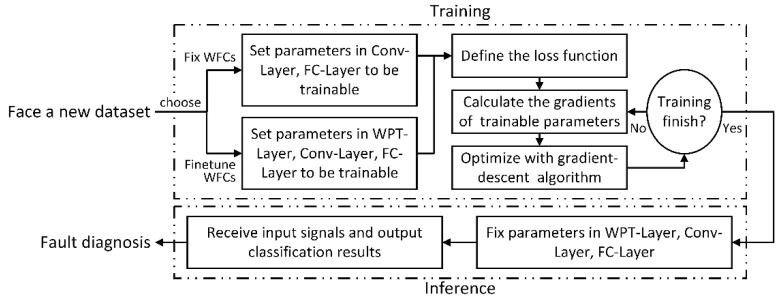
Flow chart for the training process of WPT-CNN.

In this paper, the diagnosis performance of WPT-CNN with fixing and fine-tuning the WFCs are both evaluated; the former treats WPT-Layer as a constant processing module and the latter sets the two convolutional kernels in WPT-Layer to be trainable and fine-tunes the shared WFCs along with the other parameters in the Conv-Layers and FC-Layers. Once the loss function has been defined as Equation (8), the gradients of parameters in WPT-CNN except for WPT-Layer can be calculated with standard gradient backpropagation process, while the calculation of gradient of the WFCs is rather special, following Equation (9), if WPT-Layer is set to be trainable. After the training is finished by triggering a certain predetermined condition, the model turns to fixing all parameters in the network structure, including the WFCs in WPT-Layer, and becoming ready for bearing fault diagnosis tasks. An overview of the whole training process of WPT-CNN is illustrated in Figure 6.

## 4. Experimental Results

The proposed WPT-CNN method was implemented in Python 3.7 with the DL library PyTorch and trained with a GTX1080Ti GPU. The experiments in this section used two experimental rolling bearing fault datasets to evaluate the effectiveness of the proposed method.

### 4.1. Case One: Benchmark Public Rolling Bearing Fault Dataset

#### 4.1.1. Dataset Description

**Figure 7:**
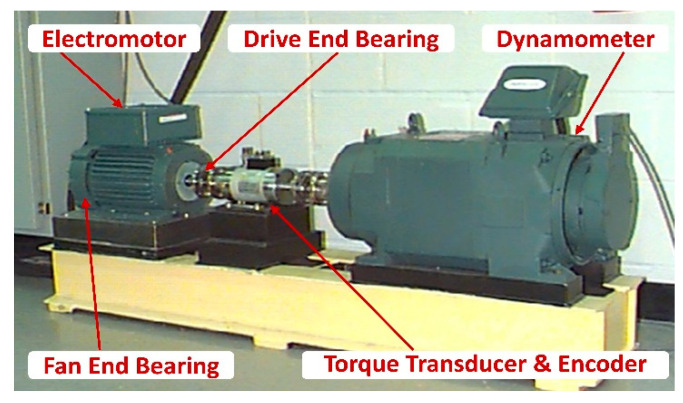
The bearing test rig.

In this case, a famous public rolling bearing dataset from the Case Western Reserve University bearing data center [44] is conducted to verify the proposed method. The experiment was built on a test stand that consisted of a 2hp motor, a torque transducer/encoder, a dynamometer and control electronics, as shown in Figure 7. The SKF6205-2RS JEM (Svenska Kullager-Fabriken, Goteborg, Sweden) deep groove ball bearings were mounted on the motor shaft and introduced with single-point faults using electro-discharge machining. Vibration data were collected by the accelerometer attached to the housing of driven end bearing and sampled at 12,000 points per second. There are four types of working conditions (0 hp + 1797 rpm, 1 hp + 1772 rpm, 2 hp + 1750 rpm and 3 hp + 1730 rpm) and each condition contains three types of bearing faults, which are ball fault, inner race fault and outer race fault, plus one health bearing condition. Each fault type contains three damage sizes, which are 0.007 inch, 0.014 inch and 0.021 inch. Overall, there were ten types of bearing conditions in total. The abbreviation label of each condition is shown in Table 1.

Considering the limited volume of the experimental data files, the starting point of each observation is picked uniformly from the raw vibration signal to minimize the data overlap. The length of each observation is selected as 1024 data points and each 500 observations are sampled from each bearing condition and each working condition. Two datasets with different working condition assignments are generated to validate the proposed fault diagnosis method. In each dataset, the total observations are spilt into the training set, the validation set and the testing set. The training set is used to train the model, and the validation set is used to prevent overfitting and indicate the training completion when accuracies get saturated. The testing set is used to evaluate the model’s final performance. Details of the two datasets are shown in Table 2.

#### 4.1.2. Hyperparameter Setup

Reasonable hyperparameter setup is important to maximize the model performance. Adam [45] optimizer has a very fast converging speed and can assign adaptive learning rate per parameter, which is selected to train the model in this experiment. In Adam, the momentum of the gradient is set to 0.9 and the momentum of the gradient square is set to 0.99, which are two good default values. The mini-batch size of each iteration is set to 32 and the weight decay is set to 0.1 to prevent overfitting and pursue better generalization. As discussed in Section 3.3, weight decay is not activated in WPT-Layer and the extra penalty term in the loss function replaces the function of weight decay. The trade-off ***α*** of the penalty term is set to 0.1.

**Figure 8:**
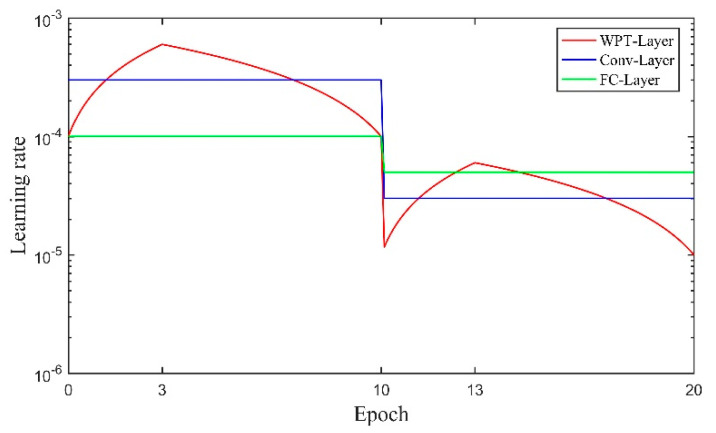
Learning rate schedules used for training different network layers in Case One.

There are three kinds of learning rate schedules for WPT-Layer, Conv-Layers and FC-Layer, respectively. Total training epoch number (during which the model is ensured to complete the training) is set to 20. The schedule of Conv-Layers is to set the initial learning rate to 0.0003, and divide it by 10 at the 10th epoch. However, the parameters in WPT-Layer are expected to explore the solution space more aggressively, and thus use a triangle learning rate schedule [46] in which learning rate begins at 0.0001 and linearly increases during the first three-tenths of the local journey up to 0.0006, then decreases back to 0.0001. To match the scale of learning rate of Conv-Layers, the learning rate for WPT-Layer is also divided by 10 at the 10th epoch. The learning rate for FC-Layer is set to 0.0001 initially and divided by 2 at the 10th epoch, because the parameters in FC-Layer are expected to optimize slightly at the beginning and explore fully at the ending (when the parameter values of the preceding layers tend to be stable). The three different learning rate schedules are shown in Figure 8.

As shown in Figure 5, m is the number of convolutional kernels in the first 2D Conv-Layer that can influence the capability of network. The setup of m must be careful because too few convolutional kernels are inadequate to handle complicated tasks while for too many kernels overfitting is easy to occur. After several comparison tests, the suitable number of m is set to 32. Dropout rate of the FC-Layer is set to 0.3.

To make the overall setups clear, Table 3 exhibits all existing hyperparameters and the corresponding setting values.

#### 4.1.3. Effectiveness of the WPT-CNN

In this part, the performance of the proposed WPT-CNN method is tested on two datasets and initialized by the filter coefficients of “db1”, “db2”, “db3” and “db4” wavelet function, respectively. The kernel length 2k of the WPT-Layer is consistently set to 8 and zeros are filled into the two ends of the kernel when initializing coefficients are inadequate. Table 4 shows the detailed parameters of the proposed WPT-CNN. For each “WPT-CNN-db*x*” model, the performance differences between fixing wavelet filter coefficients and fine-tuning the coefficients are evaluated and the two schedules share the same initializing network parameters. Detailed training schedule setups are shown in Section 4.1.2. Training processes of models last up to 20 epochs and the performances are evaluated with a validation set after each epoch. Once the validation accuracies get saturated, the trainings are considered as completed and the models are then tested on the testing set. The testing results of ten trials for each WPT-CNN model setup on two datasets are listed in Table 5.

From the results in Table 5, it can be seen which setup of the proposed method is satisfactory in both datasets. In particular, the coefficient-finetuned model always obtains superior average testing accuracies to its coefficient-fixed counterpart, which indicates that optimized WFCs are indeed effective to further improve the performance of the proposed method. The best testing average accuracies in Dataset A and Dataset B are 99.73% and 99.48%, respectively. Dataset B evaluates the robustness of the proposed method faced with limited fault data and the accuracy results are comparatively acceptable. Originally, different initialization wavelet functions have different testing performances on two datasets and it is hard to tell which one is the best choice for this specific fault task. For example, the “db1”, “db3” and “db4” wavelet functions perform similarly in Dataset A with the average testing accuracy of 99.62%, 99.64% and 99.61%, respectively, while the “db2” function has the worst accuracy of 99.47%, and the opposite happens in Dataset B, where the “db2” function performs the best with a testing accuracy of 99.43% while the “db1”, “db3” and “db4” functions perform with much inferior accuracies of 99.12%, 99.08% and 99.06%, respectively. It is worth noticing that the accuracy gaps between four functions may seem numerically similar, but these minor accuracy differences show the models’ performance limitations on hard classification samples. However, fine-tuning the WFCs leads to the accuracies of WPT-CNN falling into a much higher and more stable numerical scope, which alleviates this average testing accuracy difference issue. In Dataset A, the four coefficient-finetuned WPT-CNNs have average testing accuracies ranging from 99.70% to 99.73%, and in Dataset B, the accuracies range from 99.30% to 99.48%. These results indicate that the proposed method has found a way to ease the wavelet function selection problem, since different functions can perform similarly. Moreover, lower standard deviations show again that optimizing WFCs is worthy of consideration.

**Figure 9:**
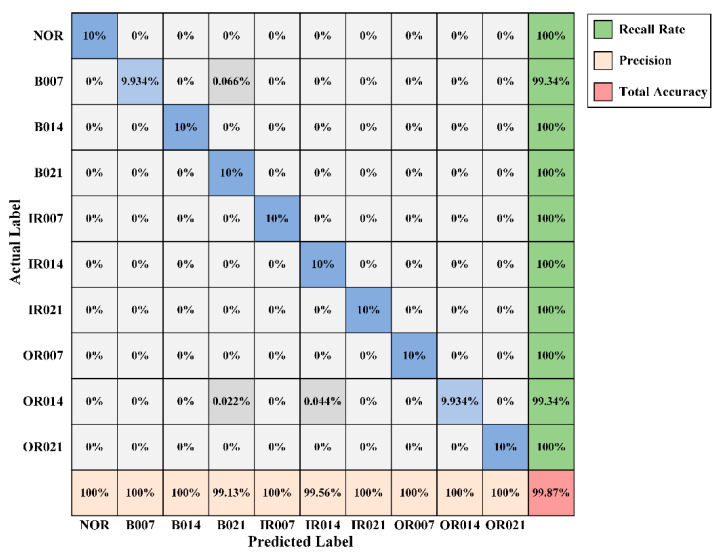
Confusion matrix of the best trial of WPT-CNN in Case One.

**Figure 10:**
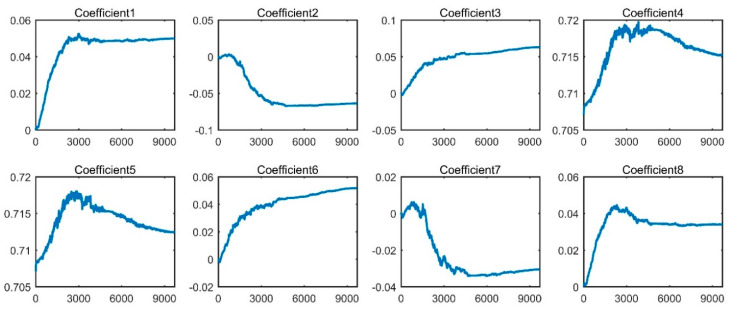
Optimizing process of the wavelet filter coefficients (WFCs) of WPT-CNN-finetuned-db1.

Figure 9 presents the confusion matrix of the best trial of WPT-CNN for Dataset A, which obtained the testing accuracy of 99.87% with the “finetuned- db1” setup. Additionally, Figure 10 demonstrates an optimizing process of the eight WFCs of this model, in which it is observed that each coefficient steadily tends to another numerical level. In the confusion matrix, rows stand for the actual label for each condition, and columns stand for the predicted label that the model gives out; in addition, the lowest row represents the precision for each condition, and the rightmost column represents the recall rate. Seen from Figure 9, NOR, B014, B021, IR007, IR014, IR021, OR007 and OR021 are perfectly classified, but B007 and OR014 are slightly misclassified. OR014 is the worst one but its recall rate still reaches 99.34% with only three error diagnosis. For the best trial of the “fixed-db1” counterpart, there are overall fifteen error diagnosis among the 4500 testing samples, which lost some accuracy due to the lack of the coefficient fine-tuning.

#### 4.1.4. Comparison with Other Methods

Three other DL methods are compared for prediction accuracy with the proposed method in this case to evaluate the performance. They are a variant of VGGNet from [35], a 2D-CNN that removes the WPT-Layer from the WPT-CNN structure, and a 1D-CNN with the kernel length of 9 [24]. The three CNNs have similar weight amounts and computation cost. VGGNet and 2D-CNN separately fold up the time-series signal into an appropriate 2D-format data matrix as input [47], while 1D-CNN directly receives raw 1D vibration signal as input. Furthermore, other ML methods including artificial neural network (ANN), support vector machine (SVM), k-nearest neighbor (KNN), random forest (RF) and AdaBoost (AB) are also introduced to compare with the proposed method. Considered that ML methods are not working with original signal data, the inputs of these ML methods are the twenty-three manually extracted time-domain and frequency-domain features referred to in [48]. All mentioned methods are trained by the same training set and tested on the same testing set from Dataset A. The four DL methods are implemented in PyTorch using the same training hyperparameters as in Section 4.1.2, and the five ML methods are implemented in the ML library Scikit-Learn. The comparison result after ten trials is presented in Table 6. The results show that the proposed method obtains the highest performance among all fault diagnosis methods.

**Figure 11:**
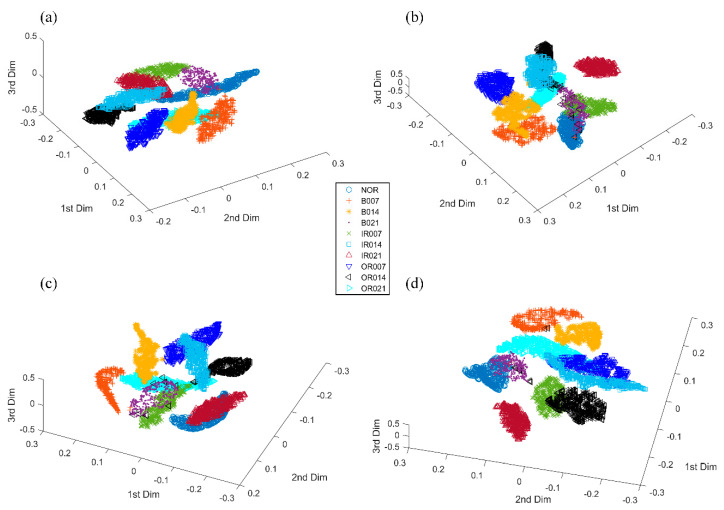
Comparison of different feature clustering maps from (**a**) WPT-CNN; (**b**) VGGNet; (**c**) 2D-CNN; (**d**) 1D-CNN in Case One.

Figure 11 displays the three-dimensional representations of high-dimensional feature maps from the final feature-extracting layer of WPT-CNN, VGGNet, 2D-CNN and 1D-CNN, respectively, by utilizing a dimensionality reduction technique known as t-distributed stochastic neighbor embedding [49] to further verify the classification performance of the proposed WPT-CNN method. Besides that, the training time and testing time of the abovementioned methods are recorded in Table 6. Three deep neural networks are trained on the GPU to accelerate training and the others are trained on an Intel i7 CPU. Since industrial machinery may not support GPU hardware, all methods are tested on the CPU to show a fair comparison. Additionally, testing time on the whole testing set is calculated. As seen in Table 7, the proposed WPT-CNN consumes more training time than any other DL and ML methods, and fine-tuning WFCs additionally greatly increases the time cost. This is because the process of 2D time-frequency matrix transformation and the corresponding gradient calculation need much computation. However, real-time computer memory usage and hardware performance will have a great impact on these specific training time and testing time values [26]. In addition, the testing time of WPT-CNN is close to that of 2D-CNN, which means the extra computation cost of WPT-Layer becomes very tiny after network weights are fixed. Less testing time of VGGNet is because of its square-sized feature maps, which enables faster matrix calculation in the computer hardware. Although it seems strange that the proposed WPT-CNN method takes the most training time among all methods, the actual testing time of WPT-CNN is not impacted and costs only 6.8 *ms* for one sample, which is acceptable for the real-time fault diagnosis of bearing components.

### 4.2. Case Two: Self-Designed Industrial Rolling Bearing Fault Dataset

#### 4.2.1. Dataset Description

**Figure 12:**
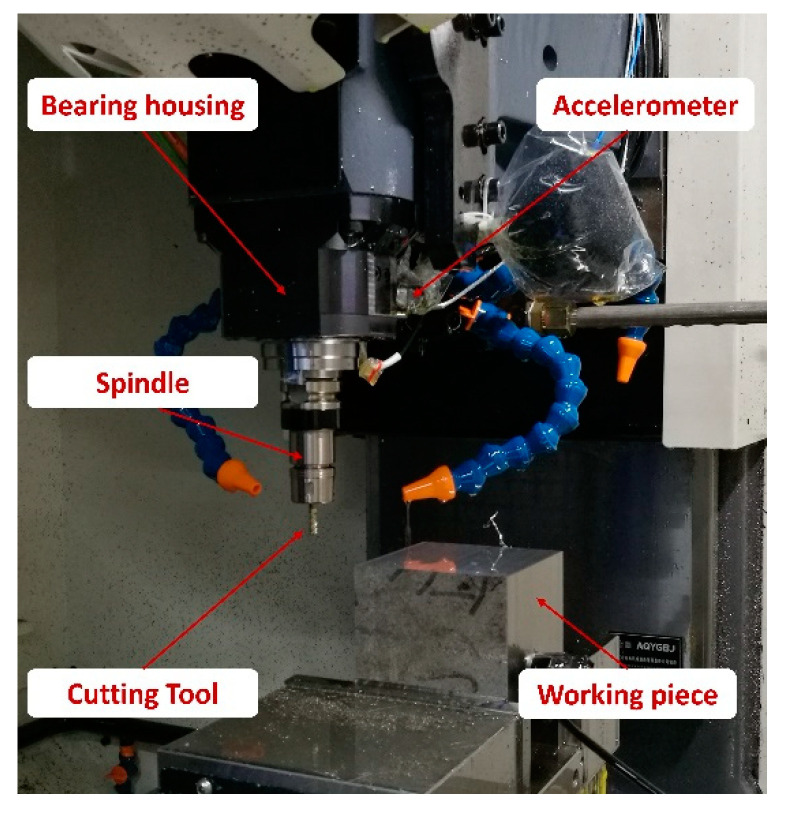
The CNC machining center test bed.

In this case, to validate the effectiveness of the proposed fault diagnosis method in realistic industrial applications, an experiment was conducted on a CNC machining center test bed to collect rolling bearing fault datasets. As shown in Figure 12, the test bed is a normal CNC machine that can achieve standard processing procedures and the lower end bearings of the spindle were replaced by the testing bearings. The testing bearings are the NSK rolling bearings of the model 40BNR10H (NSK LTD, Tokyo, Japan). A three-dimensional accelerometer (Model: Dytran 3263A2 Dytran Instruments, Inc., Chatsworth, CA, USA) was attached at the profile of a spindle box nearest to the bearing housing and an NI 9234 data acquisition card collected the vibration data at the sampling rate of 25.6 kHz.

**Figure 13:**
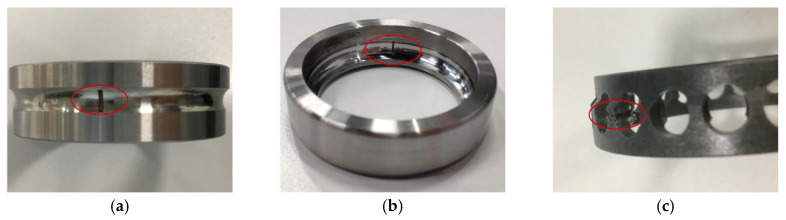
(**a**) Inner race fault; (**b**) outer race fault; (**c**) retainer broken fault.

According to the survey results on the failure causes of ninety scrapped spindle rolling bearings, this experiment designed five kinds of bearing common health states, as described in Table 8. Among the four types of fault conditions, inner race fault, outer race fault and retainer broken fault were artificially introduced using electro-discharge machining with a 600 µm width, 40 µm crack depth, and lubrication shortage fault was introduced by erasing part of the lubrication grease in the bearings. Figure 13 shows three bearings with inner race fault, outer race fault and retainer broken, respectively. To simulate the variable working conditions in realistic applications, the spindle operated in five rotating speeds and three cutting conditions for each health state, as shown in Table 9. Whenever the spindle began to rotate, a healthy cutting tool was installed on it. There were a total of five health states and fifteen working conditions in this experiment. For each health state and each working condition, the vibration signal of the rolling bearing was collected with the duration of 150 s and then saved into a separate data file.

**Figure 14:**
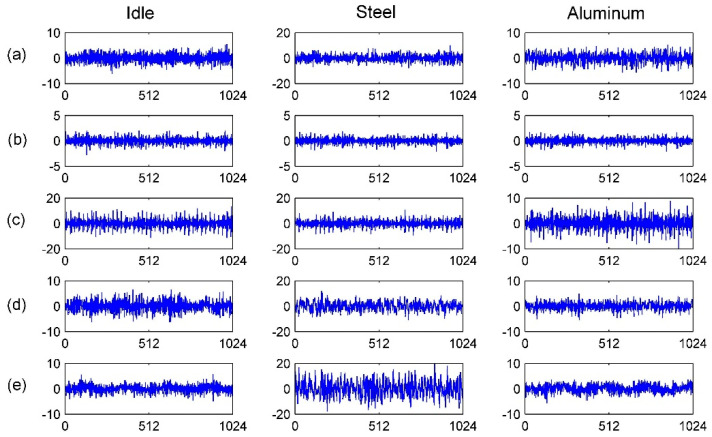
Raw vibration signals under 6000rpm and the five health states: (**a**) normal; (**b**) inner race fault; (**c**) outer race fault; (**d**) retainer broken fault; (**e**) lubrication shortage fault.

After the data acquisition, two datasets were sampled from the seventy-five data files. From each file, 1000 observations were sampled randomly and each observation contained 1024 points of vibration signal. Since the data files are large enough, this can ensure that each arbitrary two observations had no overlap and every observation was selected in a completely random order. The total number of observations of one health state was 15,000, and the total number of observations of a dataset was 75,000. Figure 14 shows the raw vibration waveforms under the five health states and it can be seen that human eyes find it hard to distinguish the signals because of strong background noise and working condition variations. To verify the robustness of the raised method, observations in the training set and the validation or testing set are from different working conditions. Details of the two datasets are shown in Table 10.

#### 4.2.2. Hyperparameter Setup

The Adam optimizer is also selected in this case due to its merits of fast converging, and so on. The moment of the gradient and the moment of the gradient square in Adam are set to 0.9 and 0.99, respectively. The mini batch is set to 32 and weight decay is set to 0.1. The trade-off of penalty term ***α*** is set to 0.1. The number of the convolutional kernels **m** is set to 32 and the dropout rate of the FC-Layer is set to 0.3. The above hyperparameter setups remain the same as in Case One.

**Figure 15:**
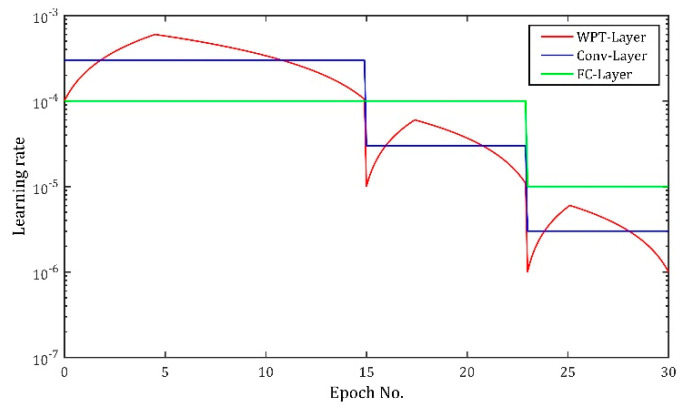
Learning rate schedules used in Case Two.

Three learning rate schedules for WPT-Layer, Conv-Layers and FC-Layer, respectively, are shown in Figure 15. First, the total training epoch number is set to 30 because larger datasets take a larger epoch number to converge. Then, the learning rate for Conv-Layers is set to 0.0003 initially, and divided by 10 at the 15th and 23rd epoch, respectively. The learning rate for WPT-Layer still adopts the triangle learning rate schedule which begins at 0.0001 and linearly increases up to 0.0006 then back to 0.0001. The learning rate for WPT-Layer is also divided by 10 at the 15th and 23rd epoch, respectively. At last, the learning rate for FC-Layer is set to 0.0001 initially and divided by 10 at the 23rd epoch for the same reason that the parameters in FC-Layer are expected to optimize slightly at the beginning and explore fully at the ending.

#### 4.2.3. Effectiveness of the WPT-CNN

The proposed WPT-CNN is conducted on the above two datasets and initialized by the filter coefficients of the “db1”, “db2”, “db3” and “db4” wavelet function, respectively. The parameters of WPT-CNN remain the same as in Case One. Testing performance differences between fixing the wavelet coefficients and fine-tuning the wavelet coefficients are also evaluated. Details of setups about training schedule are shown in the Section 4.2.2. The results of ten trials for each WPT-CNN model setup are listed in Table 11.

The results in Table 11 show which setup of the proposed method is satisfactory in both datasets, and the coefficient-finetuned model always obtains superior average testing accuracies to its coefficient-fixed counterpart, which shows that the proposed method could enjoy extra performance gains from the optimized WFCs. The best testing average accuracies in Dataset A and Dataset B are 99.80% and 99.89%, respectively. The accuracies in Dataset A are slightly lower because the testing set of Dataset A contains a rotating speed unknown to the training set, and the benefits of fine-tuning WFCs also become more evident in this more complex dataset. It is rather obvious that changing the initialization wavelet function has an impact on the final testing accuracies for the two datasets, and this influence becomes smaller when the WFCs are fine-tuned. For instance, the average accuracies of the four coefficient-fixed WPT-CNNs ranged from 99.35% to 99.64% in Dataset A, while the corresponding fine-tuned setups reduced the scope from 99.68% to 99.80%. This verifies again that the proposed method can ease the wavelet function selection problem.

**Figure 16:**
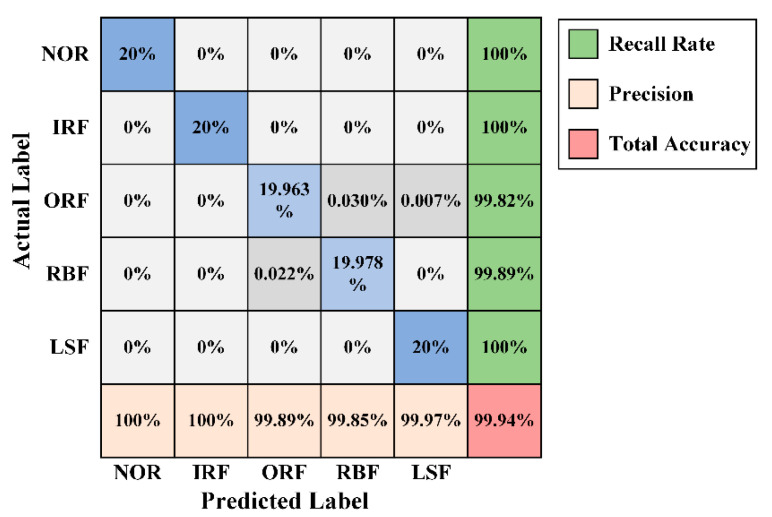
Confusion matrix of the best trial of WPT-CNN in Case Two.

**Figure 17:**
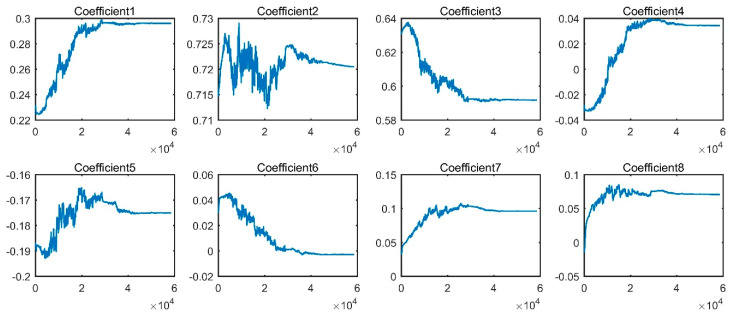
Optimization process of the WFCs of WPT-CNN-finetuned-db4.

Figure 16 presents the confusion matrix of the best trial of WPT-CNN for Dataset A, which obtained the testing accuracy of 99.94% with the “finetuned-db4” setup. Figure 17 demonstrates an optimizing process of the eight WFCs of this model. Seen from Figure 16, NOR, IRF and LSF are perfectly classified, while ORF and RBF have some slight misclassifications. ORF is the worst one but its recall rate still reaches 99.82% with only five error diagnosis. For the best trial of the “fixed-db4” counterpart, there are overall twenty-seven error diagnoses, which is nineteen more misclassifications than the best trial of “finetuned-db4”.

#### 4.2.4. Comparison with Other Methods

In this subsection, the proposed method is compared the prediction accuracy with three other DL methods and five ML methods. Three DL methods are a variant of VGGNet, a 2D-CNN and a 1D-CNN. The five ML methods include ANN, SVM, KNN, RF and AB. VGGNet and 2D-CNN separately fold up the time-series signal into an appropriate 2D-format data matrix as input, and 1D-CNN directly receives raw 1D vibration signal as input. The inputs of the five ML methods are the twenty-three manually extracted time domain and frequency domain features, remaining the same as in Case One. All mentioned methods are trained by the same training set and tested on the same testing set from Dataset A. The comparison results after ten trials are presented in Table 12. From the results, it is evident that the proposed method performs with higher accuracy than any other fault diagnosis methods.

**Figure 18:**
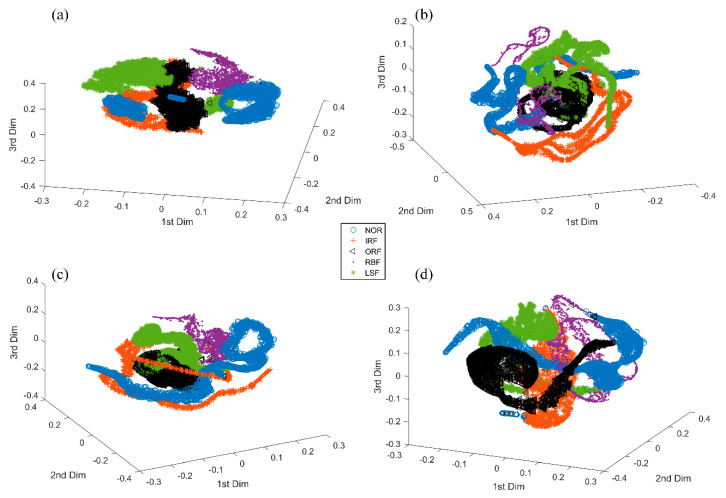
Comparison of different feature clustering maps from (**a**) WPT-CNN; (**b**) VGGNet; (**c**) 2D-CNN; (**d**) 1D-CNN in Case Two.

Figure 18 visualizes the three-dimensional representations of high-dimensional feature maps from the final feature-extracting layers of WPT-CNN, VGGNet, 2D-CNN and 1D-CNN, respectively, to verify the superiority of the proposed method in more details. The training time and testing time of above-mentioned methods are recorded in Table 13. The testing time on the whole testing set is calculated. As seen from Table 13, the proposed WPT-CNN consumes more training time than any other DL and ML methods, and fine-tuning WFCs additionally greatly increases the time cost. However, the testing time of the WPT-CNN is close to the testing time of the 2D-CNN, which means the computation cost of WPT-Layer in the testing stage is very tiny. VGGNet tests faster but has a similar training time to 2D-CNN. Although it still seems that the proposed WPT-CNN method takes the most computation time of all methods, the actual testing time of the WPT-CNN for one sample is only 6.9 ms in this case, which is acceptable for the real-time bearing fault diagnosis in industrial applications.

## 5. Discussion

This paper proposed a novel network named WPT-CNN for end-to-end fault diagnosis of rolling bearings. The WPT function is directly integrated into the network structure in the form of a modified convolutional neural layer. The overall architecture including the WPT-implementing module can be trained with the gradient backpropagation optimizing algorithm, indicating that both the time-frequency analysis and fault diagnosis capabilities of WPT-CNN are self-adaptive. Two experimental bearing fault datasets are used to validate the performance of the proposed method. Comparison results show that the WPT-CNN achieves higher accuracy and more robustness than other existing methods with original 1D signal data as input. The testing results also show that the WPT-CNN which fine-tunes WFCs along with the other network parameters can produce better performance than its coefficient-fixed counterpart, proving that the idea of automatically optimizing the time-frequency analysis capability is reasonable and promising.

Our future research work can be conducted in the following ways. First, ways to optimize the training time for the WPT-CNN could be found since fine-tuning WFCs requires a lot of computation so far in this implementing term. Second, other time-frequency analysis methods could also be studied to integrate into the neural network structures, not limited to the WPT.

## Figures and Tables

**Table 1:** Abbreviation labels of bearing conditions in Case One.

Fault Location	Damage Size (inch)			
	None	0.07	0.14	0.21
None	NOR	/	/	/
Ball	/	B007	B014	B021
Inner race	/	IR007	IR014	IR021
Outer race	/	OR007	OR014	OR021

**Table 2:** Details of the two datasets in Case One.

Working Condition	Dataset A Training/Validation/Testing	Dataset B Training/Validation/Testing
0 hp + 1797 rpm	5000/0/0	5000/0/0
1 hp + 1772 rpm	5000/0/0	5000/0/0
2 hp + 1750 rpm	5000/0/0	0/500/4500
3 hp + 1730 rpm	0/500/4500	0/500/4500
Total	15,000/500/4500	10,000/1000/9000

**Table 3:** Overviews of the hyperparameter setups.

Hyperparameter	Setup Value
Kernel number **m**	32
Decomposition level **p**	3
Dropout rate	0.3
Optimizer	Adam
Momentum	0.9; 0.99
Batch size	32
Weight decay	0.1
Trade-off ***α***	0.1
Epoch number	20
Initial learning rate	0.0001; 0.0003; 0.0001

**Table 4:** Detailed parameters of the proposed WPT-CNN.

No.	Layer Type	Kernel Size	Kernel No.	Stride	Padding	Input Size
1	WPT-Layer	8	2	1	No	1024
2	2D Conv-Layer1	3 × 3	32	1 × 1	Yes	1 × 8 × 128
3	2D Conv-Layer2	3 × 3	32	1 × 1	Yes	32 × 8 × 128
4	Max Pool-Layer1	1 × 2	N/A	1 × 2	No	32 × 8 × 128
5	2D Conv-Layer3	3 × 3	64	1 × 1	Yes	32 × 8 × 64
6	2D Conv-Layer4	3 × 3	64	1 × 1	Yes	64 × 8 × 64
7	Max Pool-Layer2	1 × 2	N/A	1 × 2	No	64 × 8 × 64
8	2D Conv-Layer5	3 × 3	128	1 × 1	Yes	64 × 8 × 32
9	2D Conv-Layer6	3 × 3	128	1 × 1	Yes	128 × 8 × 32
10	Max Pool-Layer3	1 × 2	N/A	1 × 2	No	128 × 8 × 32
11	2D Conv-Layer7	3 × 3	256	1 × 1	Yes	128 × 8 × 16
12	2D Conv-Layer8	3 × 3	256	1 × 1	Yes	256 × 8 × 16
13	Max Pool-Layer4	1 × 2	N/A	1 × 2	No	256 × 8 × 16
14	GA Pool-Layer	8 × 8	N/A	8 × 8	No	256 × 8 × 8
15	FC-Layer	N/A	N/A	N/A	No	256

**Table 5:** Testing results of different model setups on two datasets in Case One.

Model	Dataset A (%)				Dataset B (%)			
	Max	Min	Mean	Std	Max	Min	Mean	Std
fixed-db1	99.67	99.60	99.62	0.029	99.44	98.67	99.12	0.308
fixed-db2	99.71	99.31	99.47	0.188	99.64	98.84	99.37	0.327
fixed-db3	99.84	99.42	99.64	0.173	99.16	98.98	99.08	0.074
fixed-db4	99.76	99.42	99.61	0.121	99.29	98.62	99.06	0.256
finetuned-db1	99.87	99.60	99.72	0.098	99.51	99.00	99.34	0.205
finetuned-db2	99.80	99.64	**99.73**	**0.075**	99.73	99.18	**99.48**	**0.244**
finetuned-db3	99.80	99.64	99.70	0.081	99.69	99.18	99.40	0.187
finetuned-db4	99.80	99.58	99.70	0.096	99.42	98.98	99.30	0.230

**Table 6:** Comparison result on Dataset A in Case One.

Method	Average Accuracy (%)
WPT-CNN	99.73
VGGNet	98.58
2D-CNN	99.10
1D-CNN	99.25
ANN	93.78
SVM	97.23
KNN	76.13
RF	92.99
AB	60.20

**Table 7:** Cost time of different methods on Dataset A in Case One.

Method	Training Time (s)	Testing Time (s)
WPT-CNN-finetuned	236.2	30.54
WPT-CNN-fixed	165.5	28.45
VGGNet	98.3	14.85
2DCNN	137.4	29.37
1DCNN	111.4	10.65
ANN	285.2	0.20
SVM	27.1	0.35
KNN	/	0.17
RF	4.9	0.07
AB	3.1	0.07

**Table 8:** Health states of the rolling bearing in Case Two.

Health State	Label
Normal condition	NOR
Inner race fault	IRF
Outer race fault	ORF
Retainer broken	RBF
Lubrication shortage	LSF

**Table 9:** Descriptions of various working conditions.

Working Condition	Rotating Speed (rpm)	Working Piece	Feed Rate (mm/min)	Cutting Width (mm)	Cutting Height (mm)
1	6000	idle	2500	/	/
2	6000	steel 304	2500	0.3	0.1
3	6000	aluminum 7075	2500	0.3	0.1
4	7000	idle	2500	/	/
5	7000	steel 304	2500	0.3	0.1
6	7000	aluminum 7075	2500	0.3	0.1
7	8000	idle	2500	/	/
8	8000	steel 304	2500	0.3	0.1
9	8000	aluminum 7075	2500	0.3	0.1
10	9000	idle	2500	/	/
11	9000	steel 304	2500	0.3	0.1
12	9000	aluminum 7075	2500	0.3	0.1
13	10000	idle	2500	/	/
14	10000	steel 304	2500	0.3	0.1
15	10000	aluminum 7075	2500	0.3	0.1

**Table 10:** Details of the two datasets in Case Two.

Working Condition	Dataset A Training/Validation/Testing	Dataset B Training/Validation/Testing
1	0/500/4500	0/500/4500
2	0/500/4500	5000/0/0
3	0/500/4500	5000/0/0
4	5000/0/0	5000/0/0
5	5000/0/0	0/500/4500
6	5000/0/0	5000/0/0
7	5000/0/0	0/500/4500
8	5000/0/0	5000/0/0
9	5000/0/0	0/500/4500
10	5000/0/0	5000/0/0
11	5000/0/0	0/500/4500
12	5000/0/0	5000/0/0
13	5000/0/0	5000/0/0
14	5000/0/0	5000/0/0
15	5000/0/0	0/500/4500
Total	60,000/1500/13500	45,000/3000/27,000

**Table 11:** Testing results of different model setups on two datasets in Case Two.

Model	Dataset A (%)				Dataset B (%)			
	Max	Min	Mean	Std	Max	Min	Mean	Std
fixed-db1	99.57	98.96	99.35	0.255	99.89	99.72	99.82	0.073
fixed-db2	99.88	99.29	99.64	0.254	99.90	99.59	99.79	0.117
fixed-db3	99.64	98.93	99.45	0.372	99.86	99.80	99.84	0.023
fixed-db4	99.80	99.41	99.63	0.154	99.84	99.71	99.78	0.065
finetuned-db1	99.91	99.40	99.68	0.240	99.96	99.83	**99.89**	**0.055**
finetuned-db2	99.90	99.67	**99.80**	**0.084**	99.95	99.66	99.89	0.129
finetuned-db3	99.86	99.52	99.76	0.142	99.92	99.73	99.84	0.091
finetuned-db4	99.94	99.58	99.77	0.145	99.92	99.71	99.85	0.084

**Table 12:** Comparison result on Dataset A in Case One.

Method	Average Accuracy (%)
WPT-CNN	99.80
VGGNet	97.82
2D-CNN	94.31
1D-CNN	98.16
ANN	77.65
SVM	80.41
KNN	63.79
RF	65.07
AB	69.24

**Table 13:** Cost time of different methods on Dataset A in Case Two.

Method	Training Time (s)	Testing Time (s)
WPT-CNN-finetuned	1092.7	69.07
WPT-CNN-fixed	649.1	68.84
VGGNet	485.5	34.24
2DCNN	488.2	66.61
1DCNN	474.6	31.59
ANN	2191.1	1.91
SVM	275.3	0.58
KNN	/	0.68
RF	30.8	0.14
AB	14.5	0.46
